# Stress-induced hyperglycemia and mortality in patients with traumatic brain injury without preexisting diabetes: A meta-analysis

**DOI:** 10.17305/bb.2024.10865

**Published:** 2024-09-12

**Authors:** Shizhen Cui, Daiqi Xu, Han Xiong, Yimin Zhuang, Zhaohui He

**Affiliations:** 1 Department of Neurosurgery, The First Affiliated Hospital of Chongqing Medical University, Chongqing, China

**Keywords:** Stress-induced hyperglycemia, traumatic brain injury, mortality, meta-analysis

## Abstract

Stress-induced hyperglycemia (SIH) is common in patients with traumatic brain injury (TBI) and has been suggested to influence mortality rates. This meta-analysis aims to evaluate the impact of SIH on mortality in TBI patients without preexisting diabetes mellitus (DM). A comprehensive search was performed in Medline, Web of Science, Embase, Wanfang, and China National Knowledge Infrastructure (CNKI) databases up to May 15, 2024, to retrieve relevant studies. Observational studies reporting the incidence of all-cause mortality among TBI patients without preexisting DM, comparing those with and without SIH, were included. The association between SIH and all-cause mortality was analyzed using risk ratios (RR) and 95% confidence intervals (CI) with a random-effects model. Twelve cohort studies comprising 15 datasets with 16,387 TBI patients were included. The pooled analysis showed that SIH was associated with a higher risk of all-cause mortality (RR: 2.00, 95% CI: 1.72–2.33, *P* < 0.001), with mild heterogeneity (*I*^2^ ═ 25%). Sensitivity analysis confirmed the robustness of these findings. Subgroup analyses indicated no significant differences based on study design, patient age, gender proportion, SIH definition, or follow-up duration. However, the association was slightly weaker but still significant in studies using multivariate analyses (RR: 1.76) compared to univariate analyses (RR: 2.69). In conclusion, SIH was associated with a higher risk of all-cause mortality in TBI patients without preexisting DM. Further research should explore the underlying mechanisms and optimal management strategies for SIH in this population.

## Introduction

Traumatic brain injury (TBI) is a significant global health concern, often resulting from motor vehicle accidents, falls, assaults, and sports-related injuries [1, 2]. It encompasses a spectrum of severity, ranging from mild concussions to severe injuries that can lead to long-term disability or death [3, 4]. TBI affects approximately 69 million people globally each year, with varying epidemiological patterns across different regions and demographics [5]. Severe TBI, characterized by a Glasgow Coma Scale (GCS) score of 3–8, presents a complex clinical scenario with both immediate and long-term consequences [6]. Beyond the initial trauma, secondary insults, such as cerebral edema, ischemia, and inflammatory responses contribute significantly to morbidity and mortality [7, 8]. Among these secondary insults, stress-induced hyperglycemia (SIH) has emerged as a potential prognostic factor [9]. SIH, defined as elevated blood glucose levels in response to physiological stress, has been observed in a substantial proportion of TBI patients upon admission to emergency care settings [10].

The detrimental impact of SIH on TBI outcomes is attributed to its association with poorer neurological recovery and increased mortality rates [11]. Mechanistically, SIH exacerbates secondary brain injury through various pathways, including oxidative stress, mitochondrial dysfunction, and inflammation [12, 13]. These processes further compromise cerebral perfusion and neuronal function, leading to continued neurological deterioration [14]. Importantly, excluding patients with preexisting diabetes mellitus (DM) from studies assessing SIH and TBI mortality is essential, as DM independently influences outcomes following TBI [15, 16]. This exclusion helps prevent confounding in the analysis of the association between SIH and poor prognosis after TBI.

A growing number of studies have evaluated the association between SIH and prognosis after TBI [17–31]. However, the results of these studies have been inconsistent, with some not excluding patients with preexisting DM [29–31]. Therefore, in this meta-analysis, we systematically evaluated the association between SIH and all-cause mortality in TBI patients without preexisting DM. By synthesizing data from observational studies that adhered to stringent inclusion criteria, we aim to provide comprehensive insights into the impact of SIH on TBI outcomes.

## Materials and methods

This meta-analysis followed the guidelines outlined in PRISMA 2020 [32, 33] and the Cochrane Handbook for Systematic Reviews and Meta-Analyses [34], covering study design, data collection, statistical analysis, and interpretation of results. The protocol has been registered in the Open Science Framework (OSF) registry with the registration code osf.io/5gt92.

### Literature search

To identify studies relevant to the meta-analysis, we searched Medline, Web of Science, Embase, Wanfang, and China National Knowledge Infrastructure (CNKI) using comprehensive search terms, including: (1) “stress-induced hyperglycemia” OR “stress induced hyperglycemia” OR “hyperglycemia”; and (2) “traumatic brain injury” OR “traumatic brain injuries” OR “concussion” OR “traumatic cerebral injury” OR “traumatic head injury” OR “head trauma” OR “brain trauma” OR “cerebral trauma.” Key terms based on the PICOS search method are presented in Table 1. We did not limit outcomes or study design in the search terms to avoid missing potentially relevant studies. The search was restricted to clinical research involving human subjects. We only included studies published as full-length articles in English or Chinese in peer-reviewed journals. The detailed search syntax and records retrieved from each database are shown in Table S1. Additionally, we manually reviewed the references of relevant original and review articles for potentially pertinent studies. The literature published from the establishment of the databases until May 15, 2024, was considered.

**Table 1:** PICOS framework for key search terms

**Category**	**Search terms**
Population	“Traumatic brain injury” OR “traumatic brain injuries” OR “concussion” OR “traumatic cerebral injury” OR “traumatic head injury” OR “head trauma” OR “brain trauma” OR “cerebral trauma”
Intervention	“Stress-induced hyperglycemia” OR “stress induced hyperglycemia” OR “hyperglycemia”
Comparison	Non-DM patients without stress-induced hyperglycemia (not limited in search terms)
Outcomes	Mortality (not limited in search terms)
Study design	Observational studies including prospective and retrospective cohort studies, nested case-control studies, and post-hoc analyses of clinical trials (not limited in search terms)
Exclusions	NOT diabetes OR diabetic OR DM

DM: Diabetes mellitus.

### Inclusion and exclusion criteria

The inclusion criteria for potential studies were: (1) observational with longitudinal follow-up, including prospective and retrospective cohort studies, nested case-control studies, and post-hoc analyses of clinical trials; (2) included patients with TBI without preexisting DM; (3) SIH was evaluated at admission, consistent with the definition used in the original studies; (4) reported the incidence of all-cause mortality during follow-up; and (5) compared the relative risk of all-cause mortality between patients with and without SIH at baseline. Exclusion criteria included: (1) cross-sectional studies; (2) studies involving patients without TBI; (3) studies including patients with a previous history of DM; (4) studies not reporting the incidence of all-cause mortality during follow-up; or (5) studies published as conference abstracts, unpublished data, reviews, or editorials. If studies with overlapping populations were retrieved, the one with the largest sample size was included in the meta-analysis.

### Study quality evaluation and data extraction

The literature search, study identification, quality assessment, and data collection were conducted independently by two authors. In case of disagreement, the corresponding author was consulted to resolve it. To evaluate the quality of the included studies, we used the Newcastle–Ottawa Scale (NOS) [35], which assesses three aspects: selection of the population, control of confounders, and outcome measurement and analysis. NOS scores range from 1–9, with 9 indicating superior quality. We extracted various data from each study for subsequent analysis, including study information (author, year, country, and design), patient characteristics (sample size, age, sex, and GCS at baseline), the definition of SIH, the number of patients with SIH at admission, follow-up duration, the number of patients who died during follow-up, and the variables adjusted when reporting the association between SIH and all-cause mortality in the included patients.

### Statistical analysis

The relationship between SIH and the risk of all-cause mortality in TBI patients without preexisting DM was assessed using risk ratios (RR) and corresponding 95% confidence intervals (CI), comparing individuals with and without SIH at baseline. For studies reporting odds ratios (ORs), data were converted to RRs for the meta-analysis (RR ═ OR/([1-pRef] + [pRef×OR]), where pRef is the prevalence of the outcome in the reference group (non-SIH group)) [36]. RRs and their standard errors were then computed based on 95% CIs or *P* values, followed by logarithmic transformation for variance stabilization. Heterogeneity among studies was evaluated using the Cochrane *Q* test and *I*^2^ statistics [37], where an *I*^2^ > 50% indicated significant statistical heterogeneity. The findings were combined using a random-effects model to account for the influence of heterogeneity [34]. Sensitivity analyses, excluding one dataset at a time, were conducted to assess the robustness of the results. Predefined subgroup analyses were also performed to examine how study characteristics influenced outcomes, using median values of continuous variables as cutoffs for defining subgroups. Publication bias was assessed through the construction of funnel plots and visual inspection for plot symmetry [38]; an Egger’s regression test was also performed [38]. Statistical analysis was performed using RevMan (Version 5.1; Cochrane Collaboration, Oxford, UK) and Stata software (version 12.0; Stata Corporation, College Station, TX, USA).

## Results

### Study inclusion

**Figure 1.**
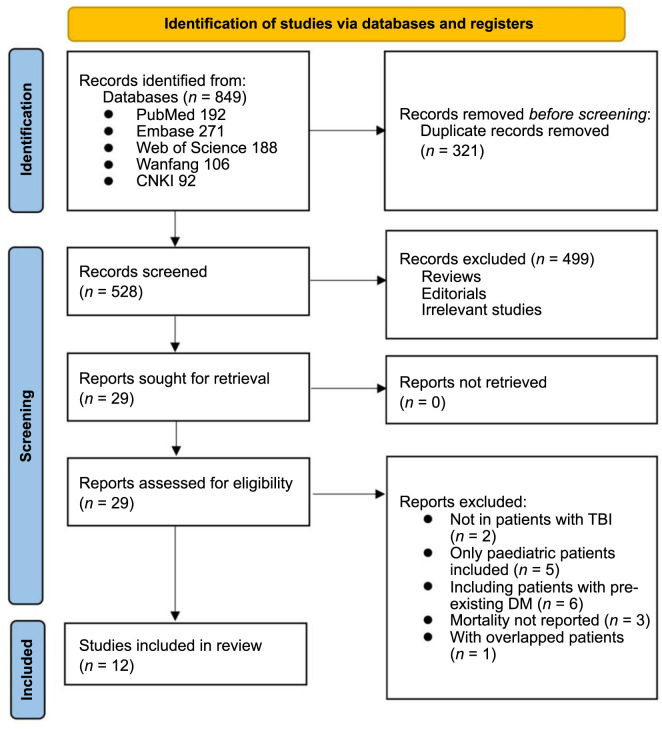
**The flowchart depicts the process of database search and study inclusion.** CNKI: China National Knowledge Infrastructure; TBI: Traumatic brain injury; DM: Diabetes mellitus.

The process of study inclusion is illustrated in Figure 1. In brief, 849 potentially relevant records were identified through a comprehensive search of the databases, of which 321 were excluded due to duplication. Subsequent screening of the remaining records, based on titles and abstracts, excluded an additional 499 studies, mostly because they were not related to the aim of the meta-analysis. Consequently, the full texts of the 29 remaining records were reviewed by two independent authors, and 17 were excluded for the reasons listed in Figure 1. Finally, 12 cohort studies were deemed suitable for quantitative analysis [17–28].

**Table 2:** Characteristics of the included studies

**Study**	**Country**	**Design**	**Sample size**	**Diagnosis**	**Bassline GCS**	**Mean age (years)**	**Men (%)**	**Definition of SIH**	**No. of patients with SIH**	**Follow-up duration (days)**	**No. of patients died**	**Variables adjusted**
Jiang, 2011	China	RC	106	TBI patients without preexisting DM and HbA1c at admission < 6%	3∼8	56.6	56.6	BG at admission ≥ 10 mmol/L	84	28	3	None
Sun, 2015	China	RC	150	TBI patients without preexisting DM	3∼8: 56.7% 9∼15: 43.3%	57.1	64.7	BG at admission ≥ 11.1 mmol/L	66	90	43	None
Bosarge, 2015	USA	RC	581	TBI patients without preexisting DM and HbA1c at admission < 6.5%	3∼8	38.4	76.2	BG at admission ≥ 11.1 mmol/L	152	During hospitalization	267	Age, sex, ISS, RTS, and lactic acid level at admission
Liu, 2015	China	RC	64	TBI patients without preexisting DM and HbA1c at admission < 6%	3∼8	54.3	78.1	BG at admission ≥ 8.3 mmol/L	42	90	21	Age, ISS, and length of ICU stay
Kafaki, 2016	Iran	PC	220	TBI patients without preexisting DM and HbA1c at admission < 6.5%	3∼8	41.4	67.7	BG at admission ≥ 11.1 mmol/L	85	During hospitalization	88	None
Khajavikhan, 2016	Iran	RC	83	TBI patients without preexisting DM	3∼8	35.3	NR	BG at admission ≥ 11.1 mmol/L	34	During hospitalization	38	None
Wang, 2019	China	RC	236	TBI patients without preexisting DM	NR	44.1	75	BG at admission ≥ 11.1 mmol/L	154	28	34	Age, hypertension, and GCS at admission
Zhang, 2020	China	RC	110	TBI patients without preexisting DM and HbA1c at admission < 6.5%	3∼8	60.9	65.5	BG at admission ≥ 11.1 mmol/L	55	28	36	Age, oxygenation index, WBC, and lactate acid at admission
Tsai, 2020	Taiwan	RC	1054	TBI patients without preexisting DM and HbA1c at admission < 6.5%	Mean: 13.7	49.8	66.7	BG at admission ≥ 11.1 mmol/L	104	During hospitalization	64	Age, sex, and GCS at admission
Tsai, 2020	Taiwan	RC	5603	TBI patients without preexisting DM and HbA1c at admission < 6.5%	Mean: 11.2	48.2	72	BG at admission ≥ 11.1 mmol/L	811	During hospitalization	429	Age, sex, and GCS at admission
Tsai, 2020	Taiwan	RC	3189	TBI patients without preexisting DM and HbA1c at admission < 6.5%	Mean: 13.3	51.8	64.7	BG at admission ≥ 11.1 mmol/L	305	During hospitalization	99	Age, sex, and GCS at admission
Tsai, 2020	Taiwan	RC	4771	TBI patients without preexisting DM and HbA1c at admission < 6.5%	Mean: 12.3	48.5	66.9	BG at admission ≥ 11.1 mmol/L	496	During hospitalization	247	Age, sex, and GCS at admission
Wang, 2020	China	RC	40	TBI patients without preexisting DM and HbA1c at admission < 6%	3∼8	42.1	70	BG at admission ≥ 11.1 mmol/L	22	180	6	None
Matovu, 2021	Uganda	PC	99	TBI patients without preexisting DM and HbA1c at admission < 6.5%	3∼8	NR	92.9	BG at admission ≥ 11.1 mmol/L	16	30	47	Age, sex, GCS at admission, temperature, and CT severity
Yu, 2023	China	RC	81	TBI patients without preexisting DM and HbA1c at admission < 6.5%	6∼8	50.3	55.6	BG at admission ≥ 11.1 mmol/L	41	28	45	Age, sex, GCS, and lactate acid at admission

BG: Blood glucose; DM: Diabetes mellitus; TBI: Traumatic brain injury; HbA1c: Hemoglobin A1c; NR: Not reported; GCS: Glasgow coma scale; SIH: Stress-induced hyperglycemia; RC: Retrospective cohort; PC: Prospective cohort; ISS: Injury severity score; RTS: Revised trauma score; ICU: Intensive care unit; WBC: White blood cell.

### Overview of study characteristics

Table 2 summarizes the characteristics of the included studies. In total, 12 cohort studies, consisting of two prospective cohorts [21, 27] and ten retrospective cohorts [17–20, 22–26, 28], were included in the meta-analysis. One study included four cohorts from different centers, which were analyzed independently [24], bringing the total number of datasets to 15. These studies were published between 2011 and 2023 and conducted in China, the United States, Iran, Taiwan, and Uganda. Overall, 16,387 TBI patients without preexisting DM were included. The mean ages of the participants ranged from 35.3 to 60.9 years, with the proportion of men ranging from 55.6% to 92.9%. The GCS were < 8 for all included patients at admission in nine studies [17–19, 21, 22, 25–28]. SIH was diagnosed as a random glucose level ≥11.1 mmol/L at admission in ten studies [18, 20–28], while two studies used thresholds of ≥8.3 mmol/L [19] and ≥10 mmol/L [17]. A total of 2467 patients (15.1%) had SIH at baseline. Follow-up durations varied from within hospitalization to six months after onset, and 1467 patients (9.0%) died during follow-up. Univariate analysis was used in five studies when reporting the association between SIH and all-cause mortality [17, 20–22, 25], while seven studies used multivariate analysis [18, 19, 23, 24, 26–28], adjusting for factors, such as age, sex, and baseline GCS. The NOS scores for the included studies ranged from six to nine stars, indicating overall moderate to good study quality (Table 3).

**Table 3:** Study quality evaluation via the Newcastle–Ottawa scale

**Study**	**Representativeness of the exposed cohort**	**Selection of the non-exposed cohort**	**Ascertainment of exposure**	**Outcome not present at baseline**	**Control for age**	**Control for other confounding factors**	**Assessment of outcome**	**Enough long follow-up duration**	**Adequacy of follow-up of cohorts**	**Total**
Jiang, 2011	0	1	1	1	0	0	1	1	1	6
Sun, 2015	0	1	1	1	0	0	1	1	1	6
Bosarge, 2015	0	1	1	1	1	1	1	1	1	8
Liu, 2015	0	1	1	1	1	1	1	1	1	8
Kafaki, 2016	1	1	1	1	0	0	1	1	1	7
Khajavikhan, 2016	1	1	1	1	0	0	1	1	1	7
Wang, 2019	0	1	1	1	1	1	1	1	1	8
Zhang, 2020	0	1	1	1	1	1	1	1	1	8
Tsai, 2020	0	1	1	1	1	1	1	1	1	8
Wang, 2020	0	1	1	1	0	0	1	1	1	6
Matovu, 2021	1	1	1	1	1	1	1	1	1	9
Yu, 2023	0	1	1	1	1	1	1	1	1	8

### Results of the meta-analysis

**Figure 2.**
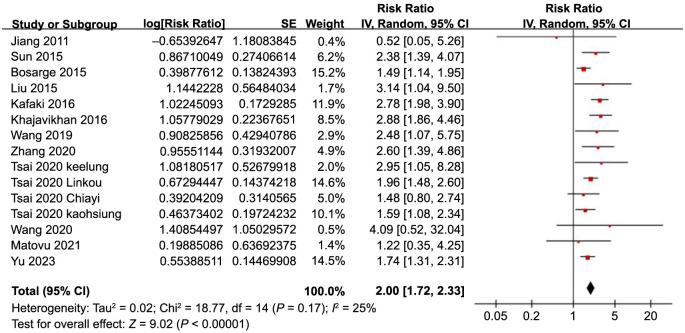
**Forest plots for the meta-analysis of the association between SIH and all-cause mortality in TBI patients without DM.** SIH: Stress-induced hyperglycemia; TBI: Traumatic brain injury; DM: Diabetes mellitus; CI: Confidence intervals.

The pooled results from 15 datasets across 12 studies [17–28] using a random-effects model suggested that SIH was associated with a higher risk of all-cause mortality in TBI patients without preexisting DM (RR: 2.00, 95% CI: 1.72–2.33, *P* < 0.001; Figure 2) with mild heterogeneity (*I*^2^ ═ 25%).

### Results of sensitivity analysis

Further analysis to assess the impact of excluding individual datasets consistently demonstrated similar results (RR: 1.88–2.09, all *P* < 0.05). Notably, sensitivity analysis limited to studies including patients with severe TBI (baseline GCS: 3–8 [17–19, 21, 22, 25–28] also produced similar results (RR: 2.10, 95% CI: 1.63–2.71, *P* < 0.001; *I*^2^ ═ 48%).

### Results of subgroup analyses

**Figure 3.**
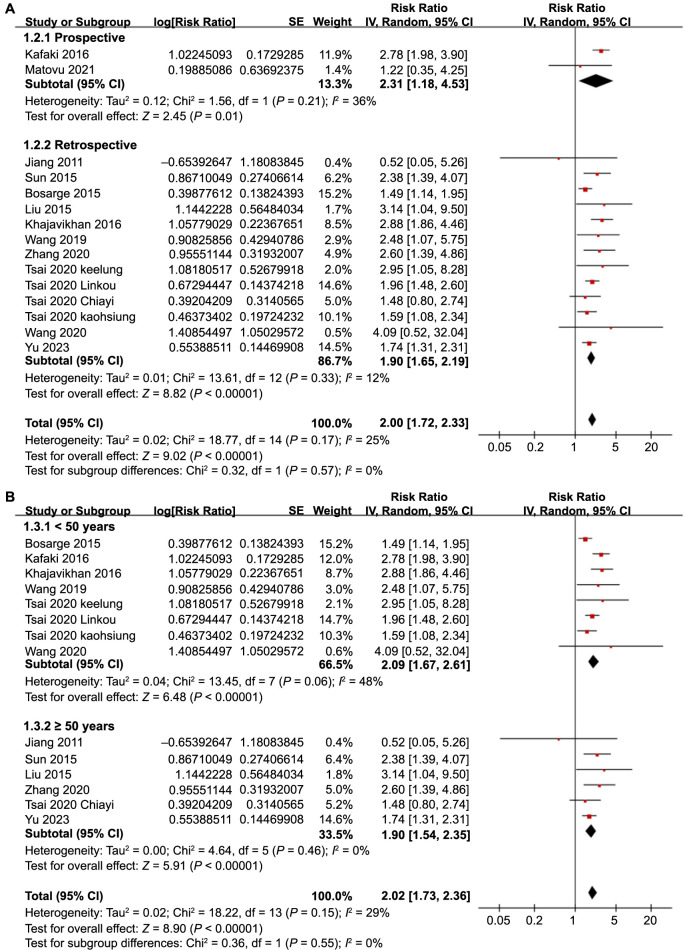
**Forest plots for the subgroup analyses of the association between SIH and all-cause mortality in TBI patients without DM.** (A) Subgroup analysis according to study design; (B) Subgroup analysis according to the mean age of the patients. SIH: Stress-induced hyperglycemia; TBI: Traumatic brain injury; DM: Diabetes mellitus; CI: Confidence intervals.

**Figure 4.**
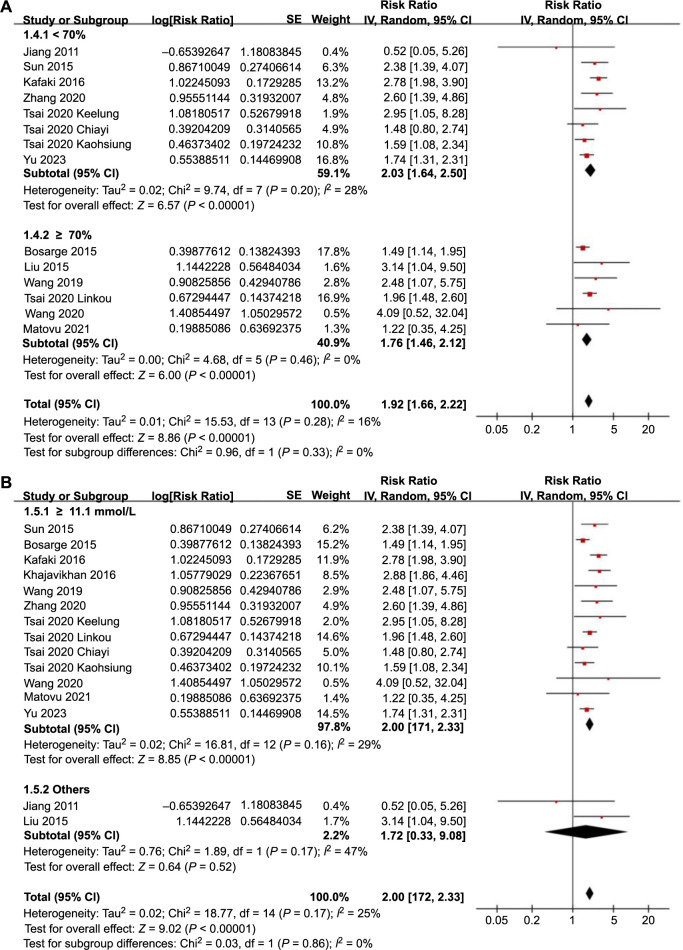
**Forest plots for the subgroup analyses of the association between SIH and all-cause mortality in TBI patients without DM.** (A) Subgroup analysis according to study proportion of men in each study; (B) Subgroup analysis according to the cutoff for defining SIH. SIH: Stress-induced hyperglycemia; TBI: Traumatic brain injury; DM: Diabetes mellitus; CI: Confidence intervals.

**Figure 5.**
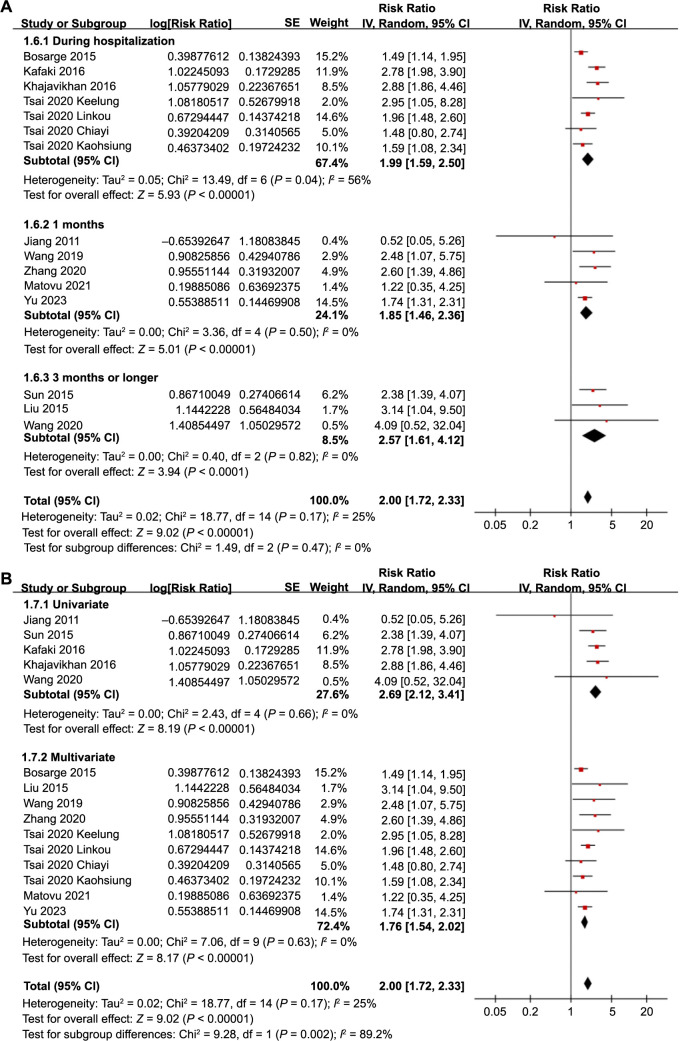
**Forest plots for the subgroup analyses of the association between SIH and all-cause mortality in TBI patients without DM.** (A) Subgroup analysis according to the follow-up duration; (B) Subgroup analysis according to the analytic models. SIH: Stress-induced hyperglycemia; TBI: Traumatic brain injury; DM: Diabetes mellitus; CI: Confidence intervals.

Further subgroup analyses showed similar results in prospective and retrospective cohorts (RR: 2.31 vs 1.90, *P* for subgroup difference ═ 0.57; Figure 3A), in studies with a mean patient age < and ≥ 50 years (RR: 2.09 vs 1.90, *P* for subgroup difference ═ 0.55; Figure 3B), and in studies with the proportion of men < and ≥ 70% (RR: 2.03 vs 1.76, *P* for subgroup difference ═ 0.33; Figure 4A). Moreover, subgroup analyses found similar results in studies with different cutoffs for defining SIH (*P* for subgroup difference ═ 0.86; Figure 4B), and in studies with different follow-up durations (*P* for subgroup difference ═ 0.47; Figure 5A). Lastly, subgroup analysis showed that the association between SIH and the risk of all-cause mortality was weakened but remained statistically significant in studies using multivariate analysis (RR: 1.76, 95% CI: 1.54–2.06, *P* < 0.001; *I*^2^ ═ 0%) compared to studies using univariate analysis (RR: 2.69, 95% CI: 2.12–3.41, *P* < 0.001; *I*^2^ ═ 0%; *P* for subgroup difference ═ 0.002; Figure 5B), which completely explained the source of heterogeneity.

### Publication bias evaluation

**Figure 6.**
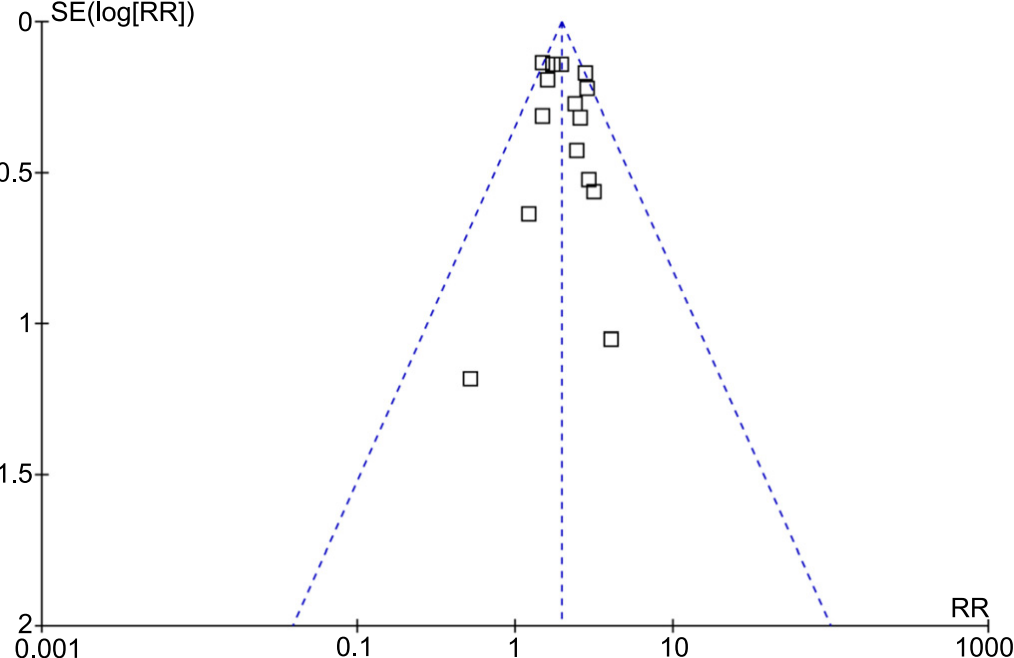
**Funnel plots for the meta-analysis of the association between SIH and all-cause mortality in TBI patients without DM.** SIH: Stress-induced hyperglycemia; TBI: Traumatic brain injury; DM: Diabetes mellitus; CI: Confidence intervals.

Visual inspection of funnel plots for the meta-analysis of the relationship between SIH and all-cause mortality in TBI patients without DM suggested symmetry, indicating a low likelihood of publication bias (Figure 6). Furthermore, Egger’s regression test results (*P* ═ 0.69) supported this conclusion, suggesting a low risk of publication bias.

## Discussion

This meta-analysis revealed a significant association between SIH and increased all-cause mortality in TBI patients without preexisting DM. The results showed a pooled RR of 2.00, underscoring the substantial impact of SIH on patient outcomes, with a consistent trend across various subgroups and sensitivity analyses. These findings suggest that SIH serves as an independent risk factor for mortality in this population, implicating its role beyond mere correlation.

The association between SIH and increased mortality risk in TBI patients can be explained through several interconnected mechanisms. Elevated blood glucose levels during acute stress, such as that induced by TBI, trigger a cascade of physiological responses that exacerbate secondary brain injury [16]. Hyperglycemia is known to induce oxidative stress by promoting the production of reactive oxygen species (ROS) and impairing antioxidant defenses [39, 40]. This oxidative stress damages cellular membranes, proteins, and DNA, contributing to neuronal cell death and worsening neurological outcomes post-TBI [41]. Additionally, hyperglycemia disrupts cerebral autoregulation, the mechanism by which the brain maintains constant blood flow despite fluctuations in systemic blood pressure [42, 43]. This dysregulation can lead to cerebral hypoperfusion or hyperperfusion, exacerbating ischemic injury or increasing the risk of cerebral edema, respectively [44].

Moreover, elevated glucose levels contribute to endothelial dysfunction and the disruption of the blood-brain barrier, facilitating neuroinflammatory responses [45]. Inflammation in the brain post-TBI is associated with microglial activation, the release of proinflammatory cytokines, and the recruitment of immune cells, which further perpetuates neurodegeneration and impairs tissue repair processes [46]. Hyperglycemia can also impair mitochondrial function, leading to energy depletion and cellular apoptosis in the injured brain tissue [47]. Mitochondrial dysfunction exacerbates neuronal vulnerability to excitotoxicity and apoptosis, accelerating neurodegeneration in TBI patients [48]. Finally, SIH has been associated with the development of coagulopathy [49] and deep venous thrombosis [50] in TBI patients, which could further worsen their prognosis. Together, these mechanisms highlight the multifaceted role of SIH in exacerbating secondary brain injury and contributing to increased mortality risk.

Findings from subgroup analyses consistently supported the primary conclusion that SIH significantly increases mortality risk in TBI patients without preexisting DM. Subgroups based on study design and patient demographics showed consistent trends, indicating robustness in the association across different populations and methodological approaches. Notably, multivariate analyses in studies examining SIH and mortality risk in TBI patients adjusted for confounders, such as age, sex, and TBI severity, revealing a slightly attenuated but still significant association. These analyses clarify that SIH independently exacerbates secondary brain injury mechanisms, such as oxidative stress and inflammation, which are critical factors in neurological recovery post-TBI [51, 52].

This study is innovative in several key aspects. To the best of our knowledge, it is the first meta-analysis to specifically exclude TBI patients with preexisting DM, thereby providing a clearer understanding of the impact of SIH on mortality in this specific population. Excluding patients with preexisting DM minimizes confounding factors, as chronic hyperglycemia in diabetic patients can affect SIH in TBI patients, adding variability to baseline metabolic states and diabetes management [53]. By excluding these patients, we isolate the specific impact of SIH on TBI mortality, enhancing the validity and reliability of our study. This approach offers a clearer assessment of SIH as an independent prognostic factor in a more homogeneous population. Moreover, we incorporated a comprehensive search strategy across five major databases, including Wanfang and CNKI, which allowed for the inclusion of studies from diverse geographical regions and populations. This broad scope enhances the generalizability of our results. Additionally, only longitudinal observational studies were included to strengthen the robustness of temporal associations between SIH and mortality in TBI patients. Lastly, our study provides detailed subgroup and sensitivity analyses, offering robust and nuanced insights into the relationship between SIH and mortality in TBI patients.

However, several limitations should be considered. Ten of the included studies were of retrospective design, which may be associated with recall and selection biases [54]. Nonetheless, subgroup analysis according to study design showed similar results. Additionally, despite efforts to adjust for confounders in multivariate analyses, residual confounding from unmeasured variables, such as comorbidities and medications cannot be entirely excluded. Variations in follow-up duration and methods for assessing mortality outcomes may also affect the interpretation and generalizability of the pooled results, warranting cautious consideration. Lastly, a causative relationship between SIH and increased mortality risk cannot be established based on our findings, as this is a meta-analysis of observational studies.

This study underscores several critical clinical implications for managing TBI patients, particularly regarding SIH. Early identification and proactive management of SIH are pivotal for improving patient outcomes. Monitoring blood glucose levels in TBI patients without preexisting DM, and timely intervention to mitigate the heightened mortality risk associated with hyperglycemia, are essential [11]. Implementing targeted interventions, such as insulin therapy or structured glucose management protocols, may potentially attenuate the adverse effects of SIH on mortality [55]. Future research should explore the mechanistic pathways linking SIH to worsened outcomes in TBI, including oxidative stress, inflammation, and impaired cerebral autoregulation. This exploration may yield tailored therapeutic strategies aimed at mitigating neurological sequelae and enhancing survival rates in TBI patients. Additionally, investigating the long-term impacts of chronic hyperglycemia on cognitive function and quality of life post-TBI is crucial for developing comprehensive management approaches. Addressing these research gaps will inform clinical practices aimed at optimizing outcomes and fostering better recovery trajectories for TBI patients affected by SIH.

## Conclusion

In conclusion, this meta-analysis consolidates evidence supporting the detrimental impact of SIH on mortality in TBI patients without preexisting DM. The findings highlight that SIH significantly increases the risk of all-cause mortality in this population. This association remained consistent across various subgroups, including different study designs, patient demographics, and definitions of SIH. The results suggest that SIH is an independent prognostic factor for mortality in TBI patients, likely due to its exacerbation of secondary brain injury mechanisms, such as oxidative stress, inflammation, and impaired cerebral autoregulation.

## Supplemental data

**Table S1:** Search syntax used in the relevant databases (last search May 15, 2024)

**Database**	**Search syntax**	**No. of records retrieved**
**PubMed**	(“stress-induced hyperglycemia”[MeSH Terms] OR “stress induced hyperglycemia”[Title/Abstract] OR hyperglycemia[MeSH Terms] OR hyperglycemia[Title/Abstract] OR “blood glucose”[Title/Abstract] OR “plasma glucose”[Title/Abstract] OR “admission glucose”[Title/Abstract] OR “SIH”[Title/Abstract] OR hyperglycemic[Title/Abstract] OR glucose[Title/Abstract]) AND (“traumatic brain injury”[MeSH Terms] OR “traumatic brain injuries”[Title/Abstract] OR concussion[MeSH Terms] OR concussion[Title/Abstract] OR “traumatic cerebral injury”[Title/Abstract] OR “traumatic head injury”[Title/Abstract] OR “head trauma”[Title/Abstract] OR “brain trauma”[Title/Abstract] OR “cerebral trauma”[Title/Abstract]) NOT (diabetes[MeSH Terms] OR diabetic[Title/Abstract] OR DM[Title/Abstract]) Filters: Humans	192
**Embase**	(‘stress-induced hyperglycemia’:ti,ab OR ‘stress induced hyperglycemia’:ti,ab OR hyperglycemia:ti,ab OR ‘blood glucose’:ti,ab OR ‘plasma glucose’:ti,ab OR ‘admission glucose’:ti,ab OR ‘SIH’:ti,ab OR hyperglycemic:ti,ab) AND (‘traumatic brain injury’:ti,ab OR ‘traumatic brain injuries’:ti,ab OR concussion:ti,ab OR ‘traumatic cerebral injury’:ti,ab OR ‘traumatic head injury’:ti,ab OR ‘head trauma’:ti,ab OR ‘brain trauma’:ti,ab OR ‘cerebral trauma’:ti,ab) NOT (diabetes:ti,ab OR diabetic:ti,ab OR DM:ti,ab) AND ([chinese]/lim OR [english]/lim) AND [humans]/lim AND [embase]/lim	271
**Web of Science**	TS ═ (“stress-induced hyperglycemia” OR “stress induced hyperglycemia” OR hyperglycemia) AND TS ═ (“traumatic brain injury” OR “traumatic brain injuries” ) NOT TS ═ (diabetes OR diabetic OR DM)	188
**Wanfang (Original in Chinese)**	(“ 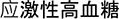 ” OR “ 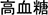 ”) AND (“ 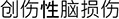 ” OR “ 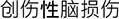 ” OR “ 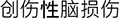 ” OR “ 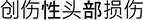 ” OR “ 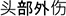 ” OR “ 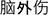 ” OR “ 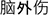 ”) NOT (“ 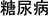 ” OR “ 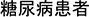 ” OR “ 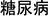 ”) Filters: 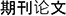	106
**Wanfang (Translation in English)**	(“stress-induced hyperglycemia” OR “hyperglycemia”) AND (“traumatic brain injury” OR “traunmatic cerebral injury” OR “traumatic cranial injury” OR “traumatic head injury” OR “head injury” OR “head trauma” OR “cerebral injury”) NOT (“diabetes” OR “diabetic” OR “diabetes mellitus”) Filters: Journal articles	106
**China National Knowledge Infrastructure (Original in Chinese)**	SU ═ (‘ 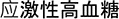 ’ OR ‘ 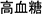 ’) AND SU ═ (‘ 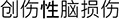 ’ OR ‘ 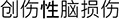 ’ OR ‘ 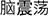 ’ OR ‘ 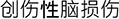 ’ OR ‘ 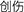 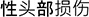 ’ OR ‘ 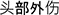 ’ OR ‘ 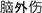 ’ OR ‘ 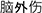 ’) NOT SU ═ (‘ 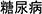 ’ OR ‘ 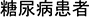 ’ OR ‘ 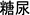 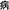 ’) Filters: 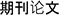	92
**China National Knowledge Infrastructure (Translation in English)**	SU ═ (“stress-induced hyperglycemia” OR “hyperglycemia”) AND SU ═ (“traumatic brain injury” OR “traunmatic cerebral injury” OR “traumatic cranial injury” OR “traumatic head injury” OR “head injury” OR “head truma” OR “cerebral injury”) NOT SU ═ (“diabetes” OR “diabetic” OR “diabetes mellitus”) Filters: Journal articles	92

## Data Availability

All the data generated during the study was within the manuscript.
